# Supplementary material for: Disorder in Mn+1AXn phases at the atomic scale
Source: Nat Commun. 2019 Feb 7;10:622. doi: 10.1038/s41467-019-08588-1 (PMC6367347; doi:10.1038/s41467-019-08588-1)
Supplement: Supplementary file 1 — Supplementary Information [file 41467_2019_8588_MOESM1_ESM.pdf]

Supplementary Information

Disorder in $M_{n+1}AX_n$ phases at the atomic scale

Wang *et al.*

Supplementary Table 1: Structural parameters for different $M_{n+1}AX_n$ phases (three 211-type phases, two 312-type phases, two 413-type phases)

211-type $M_{n+1}AX_n$ phases						
Formula	Ti_2AlC [1]		Ti_2AlN [2]		V_2AlC [3]	
Unit cell parameters (Å)	*a* = 3.06<br>*c* = 13.60		*a* = 2.99<br>*c* = 13.61		*a* = 2.91<br>*c* = 13.17	
Microstructural information	Wyckoff Notation	Atomic Positions	Wyckoff Notation	Atomic Positions	Wyckoff Notation	Atomic Positions
	Ti (4f)	(1/3, 2/3, 0.064)	Ti (4f)	(1/3, 2/3, 0.086)	V (4f)	(1/3, 2/3, 0.086)
	Al (2d)	(1/3, 2/3, 3/4)	Al (2d)	(1/3, 2/3, 3/4)	Al (2d)	(1/3, 2/3, 3/4)
	C (2a)	(0, 0, 0)	N (2a)	(0, 0, 0)	C (2a)	(0, 0, 0)

312-type $M_{n+1}AX_n$ phases				
Formula	Ti_3AlC_2 [4]		Ti_3SiC_2 [5]	
Unit cell parameters (Å)	*a* = 3.08<br>*c* = 18.58		*a* = 3.06<br>*c* = 17.65	
Microstructural information	Wyckoff Notation	Atomic Positions	Wyckoff Notation	Atomic Positions
	Ti(I) (2a)	(0, 0, 0)	Ti(I) (2a)	(0, 0, 0)
	Ti(II) (4f)	(2/3, 1/3, 0.128)	Ti(II) (4f)	(2/3, 1/3, 0.135)
	Al (2b)	(0, 0, 1/4)	Si (2b)	(0, 0, 1/4)
	C (4f)	(1/3, 2/3, 0.064)	C (4f)	(1/3, 2/3, 0.067)

413-type $M_{n+1}AX_n$ phases				
Formula	Ti4AlN3 [6]		Nb_4AlC_3 [7]	
Unit cell parameters (Å)	*a* = 2.99<br>*c* = 23.37		*a* = 3.12<br>*c* = 24.11	
Microstructural information	Wyckoff Notation	Atomic Positions	Wyckoff Notation	Atomic Positions
	Ti(I) (4f)	(1/3, 2/3, 0.054)	Nb(I) (4f)	(1/3, 2/3, 0.055)
	Ti(II) (4e)	(0, 0, 0.155)	Nb(II) (4e)	(0, 0, 0.157)
	Al (2c)	(1/3, 2/3, 1/4)	Al (2c)	(1/3, 2/3, 1/4)
	N(I) (2a)	(0, 0, 0)	N(I) (2a)	(0, 0, 0)
	N(II) (4f)	(2/3, 1/3, 0.105)	N(II) (4f)	(2/3, 1/3, 0.109)

Supplementary Table 2: Unit cell parameters and the detailed atomic position of each atom in γ-(Ti_3Al)C_2 phase. In the irradiation-induced γ-(Ti_3Al)C_2 phase, the Ti and Al cations are uniformly distributed at the cation sites with a ratio of 3:1, while the C anions are located at the cation sites with an occupancy of 0.5.

Formula	**γ-(Ti_3Al)C_2**		
Unit cell parameters (Å)	*a* = 3.05<br>*c* = 19.28		
Microstructural information	Wyckoff Notation	Site Occupancy	Atomic Positions
	Ti/Al (2a)	Ti (0.75) Al (0.25)	(0, 0, 0)
	Ti/Al (4f)	Ti (0.75) Al (0.25)	(2/3, 1/3, 0.125)
	Ti/Al (2d)	Ti (0.75) Al (0.25)	(0, 0, 1/4)
	C (4f)	C (0.5)	(1/3, 2/3, 0.0625)
	C (2b)	C (0.5)	(0, 0, 0.1875)

Supplementary Table 3: Unit cell parameters of the fcc-$(M_{n+1}A)X_n$ solid solutions. The *a* unit cell parameters of the irradiation-induced fcc-structured $(M_{n+1}A)X_n$ solid solutions were determined from the indexing and the position of the Bragg peaks, which are (111), (200), and (220), in the GIXRD patterns using the software Unitcell. [8] These unit cell parameters were also determined by measuring the average *d*-spacing along (111) in the HRTEM images and the corresponding SAED patterns and the relaxed atomic structures from the first-principles calculations. In general, the results obtained using each method are consistent with those using others in each fcc solid solution phase.

***a* unit cell parameter (nm)**	**fcc-$(M_{n+1}A)C_n$ carbides**								**fcc-$(Ti_{n+1}Al)N_n$ nitrides**		
	fcc-$(Ti_2Al)C$	fcc-$(Ti_3Al)C_2$	fcc-$(Ti_3Si)C_2$	fcc-TiC[9]	fcc-$(V_2Al)C$	fcc-VC[10]	fcc-$(Nb_4Al)C_3$	fcc-NbC[11]	fcc-$(Ti_2Al)N$	fcc-$(Ti_4Al)N_3$	fcc-TiN
**XRD**	0.421(2)	0.425(1)	0.426(2)	0.433	0.401(1)	0.416	0.436(2)	0.446	0.411(3)	0.417(2)	0.424
**HRTEM**	0.421(1)	0.423(1)	0.425(2)	-	0.403(1)	-	0.433(3)	-	0.413(2)	0.418(1)	-
**SAED**	0.422(1)	0.425(2)	0.426(2)	-	0.402(1)	-	0.434(2)	-	0.414(2)	0.418(2)	-
Ab initio	0.418	0.426	0.420	-	0.400	-	0.438	-	0.414	0.419	-

Supplementary Table 4. Calculated elastic constants of the fcc-$(M_{n+1}A)X_n$ phases. (Unit: GPa)

Phase	$\overline{C_{11}}$	$\overline{C_{12}}$	$\overline{C_{44}}$	B	G	E
fcc-$(Ti_2Al)C$	224.9	89.8	61.3	134.9	63.8	165.3
fcc-$(Ti_2Al)N$	215.8	103.2	68.9	140.7	63.9	166.5
fcc-$(V_2Al)C$	284.8	117.4	70.7	173.3	75.9	198.6
fcc-$(Ti_3Al)C_2$	264.4	96.5	78.9	152.5	80.9	206.2
fcc-$(Ti_3Si)C_2$	300.5	115.2	88.8	177.0	90.4	231.6
fcc-$(Ti_4Al)N_3$	285.1	133.8	109.1	184.2	95.7	244.8
fcc-$(Nb_4Al)C_3$	296.3	151.8	83.4	200.0	78.9	209.3

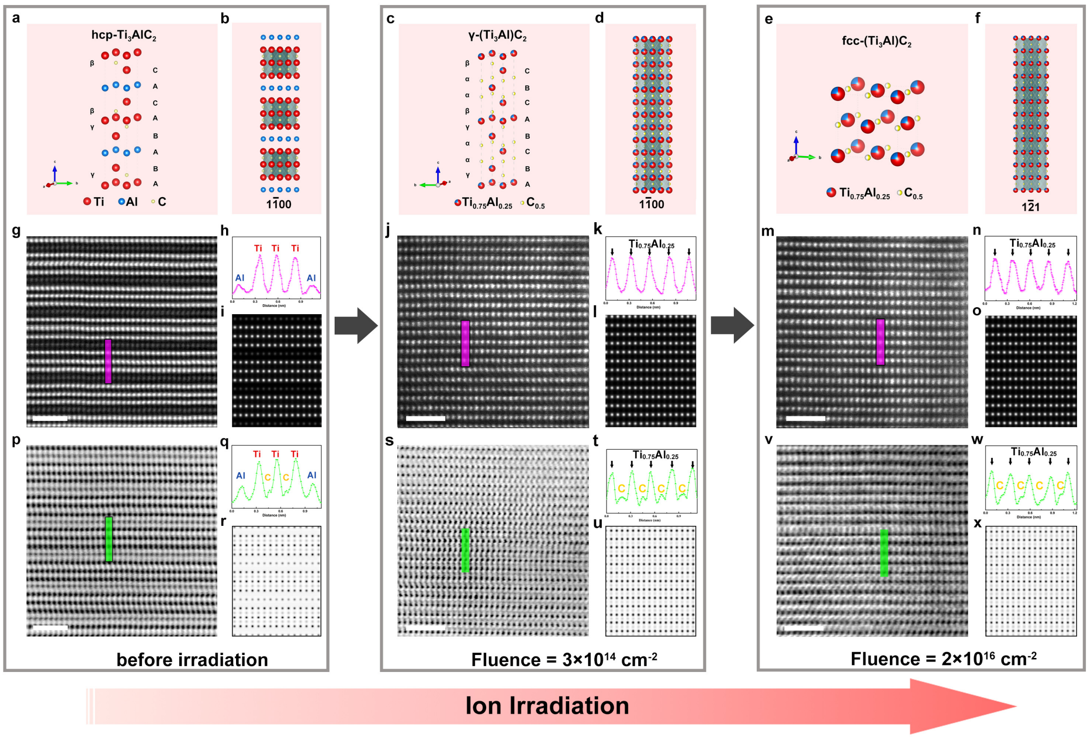

Supplementary Figure 1 | Structural models and STEM results for Ti_3AlC_2 before and after irradiation along $[1\bar{1}00]$. Schematic and the corresponding atomic arrangements along $[1\bar{1}00]$ of pristine hex-Ti_3AlC_2 (**a-b**), as well as γ-$(Ti_3Al)C_2$ (**c-d**) and fcc-$(Ti_3Al)C_2$ (**e-f**) induced by ion irradiation at the fluence of 3×10^{14} and 2×10^{16} cm^{-2}, respectively. The capital letters and the Greek letters in **a** and **c** represent the stacking sequences of the cations and anions, respectively. The octahedra in **b**, **d**, and **f** indicated that the anions are located at the octahedral interstitial sites of the cations. The crystallographic relationship between the hex-$(Ti_3Al)C_2$, γ-$(Ti_3Al)C_2$ and fcc-$(Ti_3Al)C_2$ is $[1\bar{1}00]hex // [1\bar{2}1]fcc$. (**g**, **j**, and **m**) STEM HAADF images of hex-Ti_3AlC_2, γ-$(Ti_3Al)C_2$ and fcc-$(Ti_3Al)C_2$. The contrast profiles along the purple lines are shown in **h**, **k**, and **n**, which indicates the solid solution process of the Ti/Al atoms at the cation sites. (**i**, **l**, and **o**) Simulated STEM HAADF images of hex-Ti_3AlC_2, γ-$(Ti_3Al)C_2$ and fcc-$(Ti_3Al)C_2$, which agree well with the experimental results in **g**, **j**, and **m**, respectively. (**p**, **s**, and **v**) STEM ABF images of hex-Ti_3AlC_2, γ-$(Ti_3Al)C_2$ and fcc-$(Ti_3Al)C_2$. The contrast profiles along the green lines are shown in **q**, **t**, and **w**, which indicates the atomic rearrangements of C atoms at the anion sites. The contrast is inverted for a convenient visualization. (**r**, **u**, and **x**) Simulated STEM ABF images of hex-Ti_3AlC_2, γ-$(Ti_3Al)C_2$ and fcc-$(Ti_3Al)C_2$, which agree well with the experimental results in **p**, **s**, and **v**, respectively. The scale bars on the HAADF and ABF images correspond to 1 nm. In the pristine sample, the different contrast of the Ti layers and Al layers were observed in the HAADF, indicating the ordered arrangement of the cations. The C anions are located at the octahedral sites between the Ti atoms. After irradiation to fluence of 3×10^{14} cm^{-2} and 2×10^{16} cm^{-2}, the cation contrast in HAADF/ABF images (Supplementary Figure 1(j, s, m, v)) and the *d*-spacings along [0001] become identical, indicating uniform arrangement of the cations and corresponding rearrangement of the

anions. It should be noted that the cation periodicity of the hexagonal phase and the irradiation-induced fcc phase cannot be distinguished along $[1\bar{1}00]$. However, the accuracy of the structural analysis can be enhanced by considering the atomic rearrangements from two different orientations.

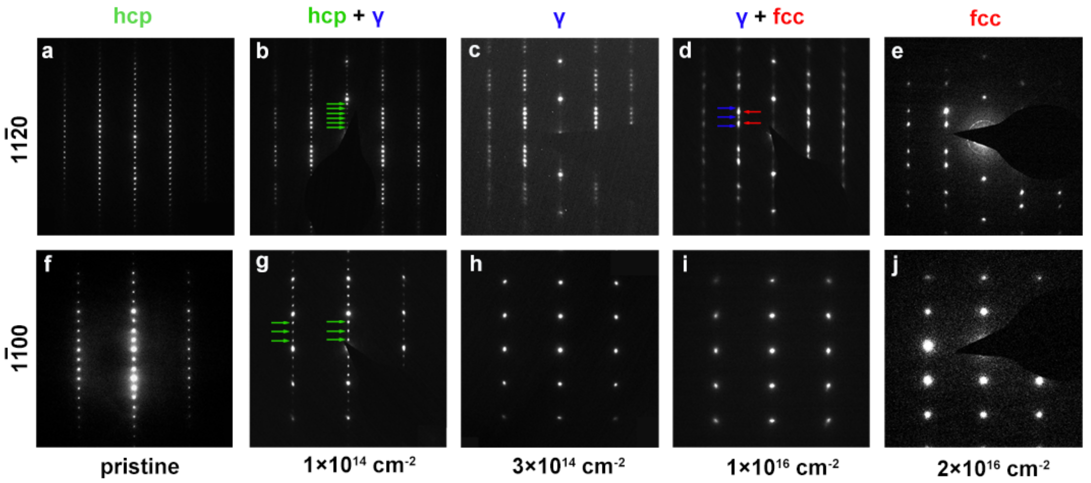

Supplementary Figure 2 | Structural modification of Ti_3AlC_2 induced by ion irradiation. Electron diffraction patterns of Ti_3AlC_2 samples before (**a**, **f**) and after irradiation at the fluence of (**b**, **g**) 1×10^{14}, (**c**, **h**) 3×10^{14}, (**d**, **i**) 1×10^{16} and (**e**, **j**) 2×10^{16} cm^{-2}, respectively. The electron beam is parallel to $[11\bar{2}0]$ (**a-e**) and $[1\bar{1}00]$ (**f-j**), respectively. The hex-γ-fcc phase transformation gradually occurs as the fluence increases. The green arrows in (**b**, **g**) indicate the hexagonal phase and the red arrows in (**d**, **i**) indicate the fcc phase. Compared to the diffraction patterns of the pristine sample, the $(000l)$ ($l \neq 8n$) and $(\bar{1}10l)$ ($l = 8n$, $8n\pm1$) diffraction spots along $[11\bar{2}0]$ as well as the $(000l)$ ($l \neq 8n$) and $(11\bar{2}l)$ ($l \neq 8n$) spots along $[1\bar{1}00]$ significantly attenuate after irradiation to an ion fluence of 1×10^{14} cm^{-2}, and totally disappear at a fluence of 3×10^{14} cm^{-2}. This is attributed to the phase transformation from the hexagonal phase to the γ phase. As the fluence increases to 1×10^{16} cm^{-2}, there appear two sets of new diffraction spots (indicated by the red arrows in Supplementary Figure 2d), indicating the formation of a nano-twinned fcc structure. At 2×10^{16} cm^{-2}, this newly formed fcc phase becomes dominant, as shown in Supplementary Figure 2 (e, j).

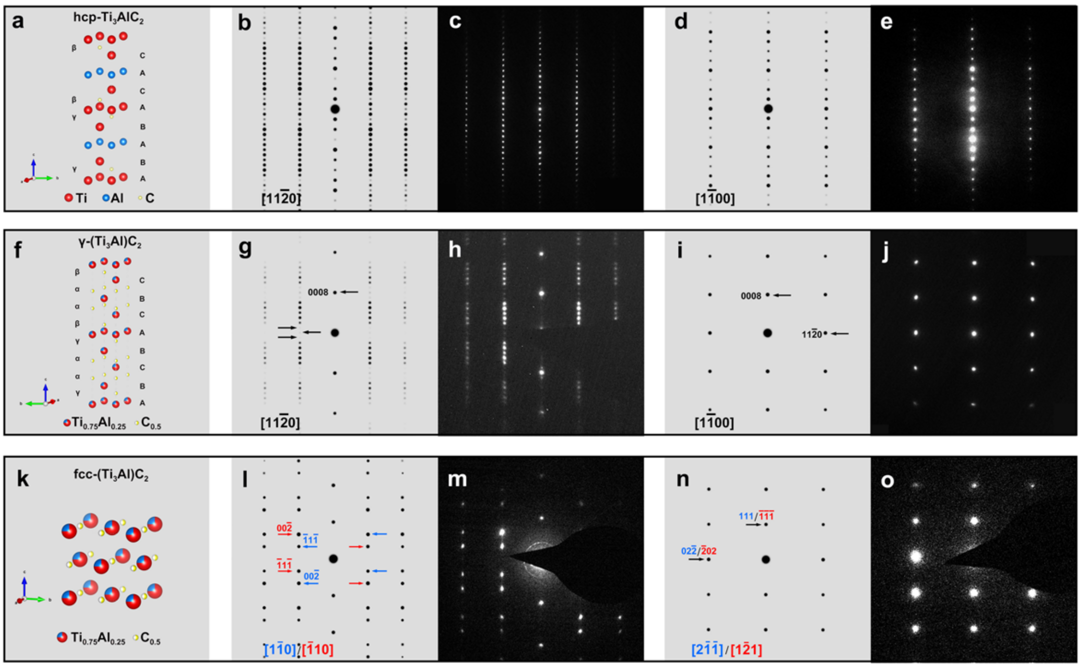

Supplementary Figure 3 | Structure of hex-Ti_3AlC_2, γ-$(Ti_3Al)C_2$, and fcc-$(Ti_3Al)C_2$. Schematic of pristine hex-Ti_3AlC_2 (**a**), as well as γ-$(Ti_3Al)C_2$ (**f**) and fcc-$(Ti_3Al)C_2$ (**k**) induced by ion irradiation at the fluence of 3×10^{14} and 2×10^{16} cm^{-2}, respectively. (**b**, **g**, and **l**) Simulated electron diffraction patterns along $[11\bar{2}0]$ of **a**, **f**, and along $[1\bar{1}0]$(or $[\bar{1}10]$) of twinned structural **k**, which agree well with the experimental SAED patterns in **c**, **h**, and **m**, respectively. Some forbidden diffraction spots (000*l*) (*l* = odd) appear in **c** compared to **b**, which results from double diffraction. (**d**, **i**, and **n**) Simulated electron diffraction patterns along $[1\bar{1}00]$ of **a**, **f**, and along $[2\bar{1}\bar{1}]$(or $[1\bar{2}1]$) of **k**, which agree well with the experimental SAED patterns in **e**, **j**, and **o**, respectively. The transformed fcc-$(Ti_3Al)C_2$ phase exhibits a specific crystallographic relationship with the γ-$(Ti_3Al)C_2$ phase: $[11\bar{2}0]\gamma$ // $[1\bar{1}0]$(or $[\bar{1}10]$)*fcc* and $[1\bar{1}00]\gamma$ // $[2\bar{1}\bar{1}]$(or $[1\bar{2}1]$) *fcc*.

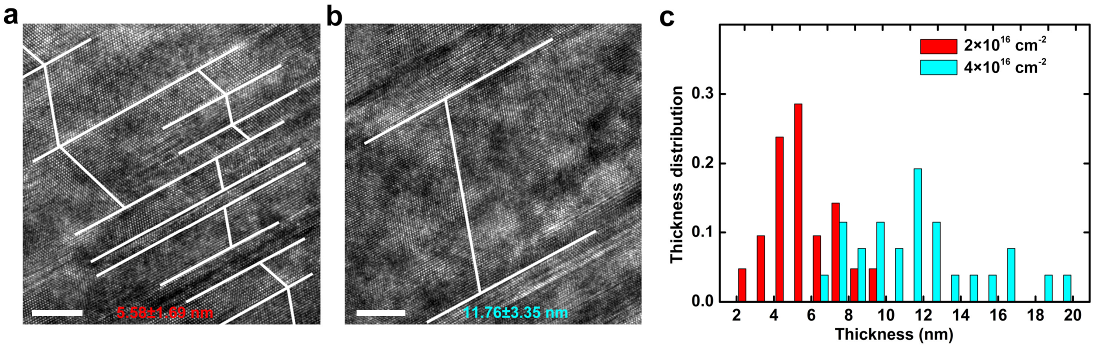

Supplementary Figure 4 | Growth of the fcc-(Ti_3Al)C_2 solid solution. High-resolution TEM images of Ti_3AlC_2 irradiated to the ion fluence of 2×10^{16} cm^{-2} (**a**) and 4×10^{16} cm^{-2} (**b**), respectively. The white lines indicate the nano-twinned structure of the fcc-(Ti_3Al)C_2 solid solution. (**c**) Thickness distribution of the nano-twinned fcc phase along [111] direction in **a** and **b**, respectively. The scale bars on the HRTEM images correspond to 5 nm. In the sample irradiated to an ion fluence of 2×10^{16} cm^{-2}, the thickness of the nano-twinned fcc phase along the [111] direction is 5.58±1.69 nm. As the fluence increases to the highest fluence achieved in our study, 4×10^{16} cm^{-2}, the thickness increases to 11.76±3.35 nm. This indicates the growth of the fcc solid solution with increasing fluence.

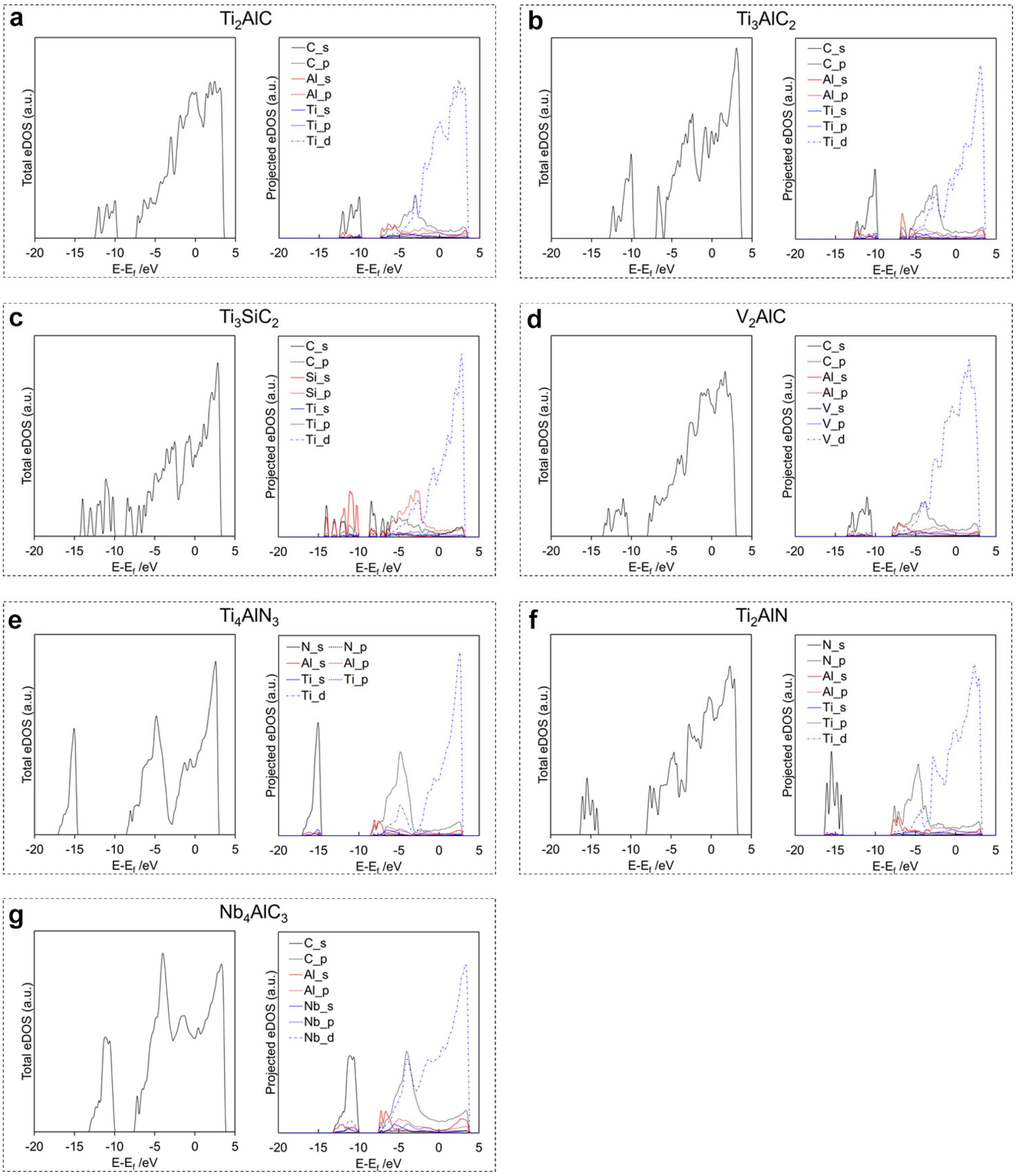

Supplementary Figure 5 | Total electronic density of state (eDOS) and projected partial eDOS of the MAX phases. (a) Ti_2AlC; (b) Ti_3AlC_2; (c) Ti_3SiC_2; (d) V_2AlC; (e) Ti_4AlN_3; (f) Ti_2AlN; (g) Nb_4AlC_3. The *s* orbitals of the X elements (i.e. C and N) are generally localized at lower energy states and have an energy gap with the *d* orbitals of the M elements (i.e. Ti and Nb). In addition, it is found that the *s/p* orbitals of the A elements (i.e. Al and Si) and the p orbitals of the X elements (i.e. C and N) are strongly hybridized with the *d* orbitals of the M elements (i.e. Ti and Nb).

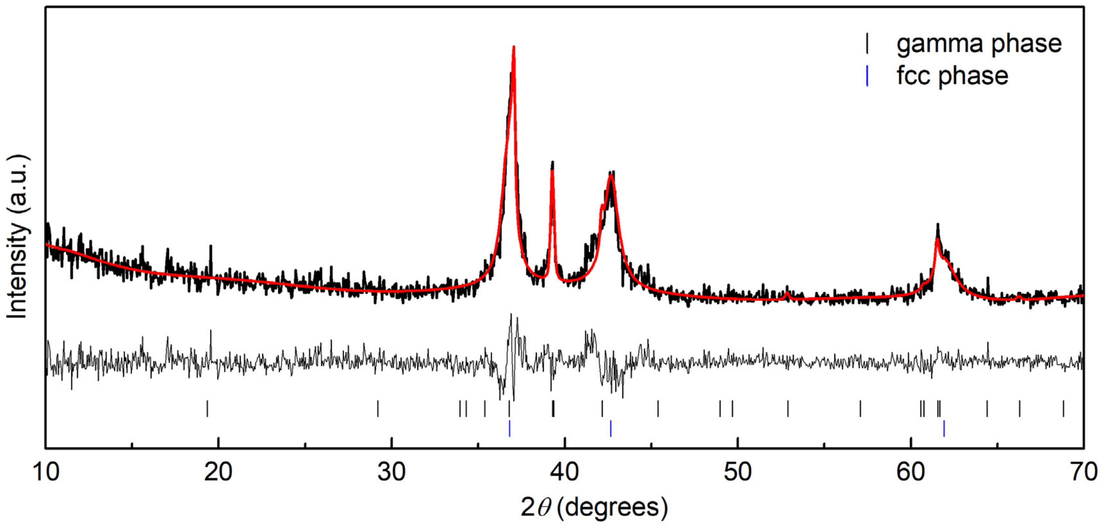

Supplementary Figure 6 | Result of Rietveld refinement. Rietveld refinement of XRD data of Ti_3AlC_2 irradiated to an ion fluence of 2×10^{16} cm^{-2}. The black line, red line and green lines at the bottom, represent the observed data, calculated result and the difference between the two, respectively. Rietveld refinement of the diffractiongrams was performed using software GSAS-II [12] to characterize hex-to-fcc phase transformations and to determine the phase ratios. The parameters refined here were unit cell parameters, scale factor, peak shape function coefficients, site occupancies, background coefficients, atomic positions, *etc*. Here, R_{wp} value for the refinement was 0.0241, while R_p value was 0.0177 and χ^2 was 9.615. The phase fractions of γ phase and fcc phase determined from this refinement are 19.1% and 80.9%, respectively, indicating that the fcc phase is dominant at 2×10^{16} cm^{-2}. Additionally, the *a* unit cell parameter determined from the refinement is 0.424 nm. These representative results of Rietveld refinements are consistent with the TEM and APT results shown in the study.

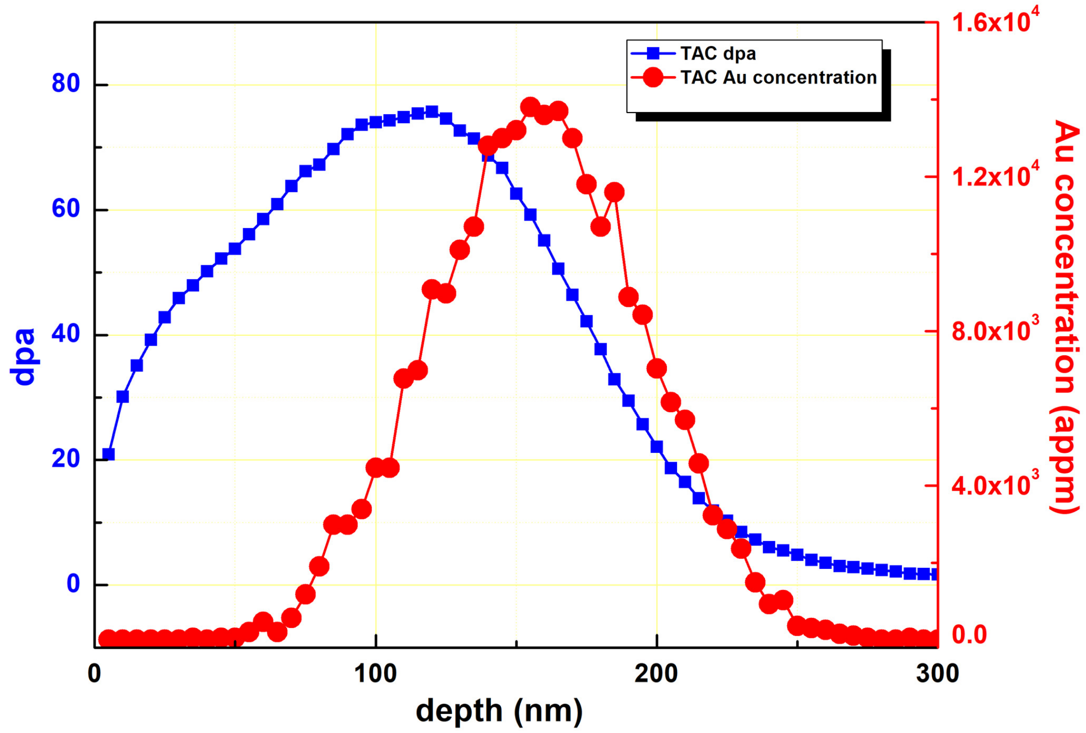

Supplementary Figure 7 | Damage profile in Ti_3AlC_2. Depth profiles of damage level, expressed in displacements per atom (dpa), and implanted ion concentration induced by 1 MeV Au^+ ion irradiation at 1×10^{16} cm^{-2} in Ti_3AlC_2. These data were calculated using the SRIM code.

Supplementary Methods:

First-principles calculations. The calculations were based on the efficient stress-strain method [13, 14]. Specifically, independent sets of strains were applied to deform the supercells of the special quasirandom structure (SQSs) in the elastic range. For a given set of strains, $\varepsilon_i = (\varepsilon_{i,1}, \varepsilon_{i,2}, \cdots, \varepsilon_{i,6})$, lattice vectors of the strained supercell, **R'**, in Cartesian coordinates can be calculated as,

Equation 1
$$\mathbf{R}' = \mathbf{D}\mathbf{R} = \begin{pmatrix} 1+\varepsilon_{i,1} & \varepsilon_{i,6}/2 & \varepsilon_{i,5}/2 \\ \varepsilon_{i,6}/2 & 1+\varepsilon_{i,2} & \varepsilon_{i,4}/2 \\ \varepsilon_{i,5}/2 & \varepsilon_{i,4}/2 & 1+\varepsilon_{i,3} \end{pmatrix} \cdot \begin{pmatrix} a_1 & a_2 & a_3 \\ b_1 & b_2 & b_3 \\ c_1 & c_2 & c_3 \end{pmatrix}$$

where **R** are the lattice vectors of the unstrained supercell. The stresses in response to the given strains are calculated via the aforementioned first-principles approach. Generally, in order to calculate the complete elastic stiffness constants, six independent sets of strains are applied on the structure:

Equation 2
$$\begin{pmatrix} \varepsilon_{1,1} & & \varepsilon_{1,6} \\ \varepsilon_{2,1} & & \varepsilon_{2,6} \\ \varepsilon_{3,1} & \cdots & \varepsilon_{3,6} \\ \varepsilon_{4,1} & & \varepsilon_{4,6} \\ \varepsilon_{5,1} & & \varepsilon_{5,6} \\ \varepsilon_{6,1} & & \varepsilon_{6,6} \end{pmatrix} = \begin{pmatrix} x & & & & & \\ & x & & & & \\ & & x & & & \\ & & & x & & \\ & & & & x & \\ & & & & & x \end{pmatrix}$$

where $x = \pm 0.01$ in the present work, and the strain components not shown are zero. Using the six sets of corresponding stresses from first-principles, the elastic stiffness constants at a fixed volume, $c_{ij}(V)$, can be determined based on Hooke's law as follows:

Equation 3
$$\begin{pmatrix} c_{11} & c_{12} & c_{13} & c_{14} & c_{15} & c_{16} \\ c_{21} & c_{22} & c_{23} & c_{24} & c_{25} & c_{26} \\ c_{31} & c_{32} & c_{33} & c_{34} & c_{35} & c_{36} \\ c_{41} & c_{42} & c_{43} & c_{44} & c_{45} & c_{46} \\ c_{51} & c_{52} & c_{53} & c_{54} & c_{55} & c_{56} \\ c_{61} & c_{62} & c_{63} & c_{64} & c_{65} & c_{66} \end{pmatrix} = \begin{pmatrix} \sigma_{1,1} & & \sigma_{1,6} \\ \sigma_{2,1} & & \sigma_{2,6} \\ \sigma_{3,1} & \cdots & \sigma_{3,6} \\ \sigma_{4,1} & & \sigma_{4,6} \\ \sigma_{5,1} & & \sigma_{5,6} \\ \sigma_{6,1} & & \sigma_{6,6} \end{pmatrix} \cdot \begin{pmatrix} \varepsilon_{1,1} & & \varepsilon_{1,6} \\ \varepsilon_{2,1} & & \varepsilon_{2,6} \\ \varepsilon_{3,1} & \cdots & \varepsilon_{3,6} \\ \varepsilon_{4,1} & & \varepsilon_{4,6} \\ \varepsilon_{5,1} & & \varepsilon_{5,6} \\ \varepsilon_{6,1} & & \varepsilon_{6,6} \end{pmatrix}^{-1}$$

Since the supercells of the SQSs used in the present work do not have a cubic symmetry, the average values of the elastic constants for the conventional cubic structures of the fcc MAX phases were derived using a similar way as employed in the Voigt-Reuss-Hill approach,

Equation 4
$$\overline{C_{11}} = (c_{11} + c_{22} + c_{33})/3$$
$$\overline{C_{12}} = (c_{12} + c_{13} + c_{23})/3$$
$$\overline{C_{44}} = (c_{44} + c_{55} + c_{66})/3$$

where $\overline{C_{11}}$, $\overline{C_{12}}$ and $\overline{C_{44}}$ are the average for the conventional cubic structures of the fcc MAX phases, and the c_{ij} are the elastic constants of the corresponding SQS obtained based on Eq. 3 from the first-principles calculations.

Based on the averaged elastic constants, aggregate properties of the bulk (*B*), shear (*G*),

and Young's (E) moduli associated with polycrystals were estimated by means of the Voigt approximation [15]; this provides the upper bound of elastic properties in terms of uniform strains. For a cubic crystal,

Equation 5 $$B = (\overline{C_{11}} + 2\overline{C_{12}})/3$$

Equation 6 $$G = (\overline{C_{11}} - \overline{C_{12}} + 3\overline{C_{44}})/5$$

Equation 7 $$E = 9BG/(3B + G)$$

The calculated elastic constants and moduli of each MAX phase in the fcc structure are summarized in Supplementary Table 4. In addition, all the investigated MAX phases have elastic constants that stratify the "Born stability criteria" (i.e. $\overline{C_{11}} - \overline{C_{12}} > 0$, $\overline{C_{11}} + 2\overline{C_{12}} > 0$ and $\overline{C_{44}} > 0$) [16], indicating they are all mechanically stable in the unstressed state in response to perturbation by elastic strains.

Supplementary References:

1. Zhou WB, Mei BC, Zhu JQ, Hong XL. Rapid synthesis of Ti_2AlC by spark plasma sintering technique. *Materials Letters* 2005, **59**(1)**:** 131-134.

2. Lin ZJ, Zhuo MJ, Li MS, Wang JY, Zhou YC. Synthesis and microstructure of layered-ternary Ti_2AlN ceramic. *Scripta Materialia* 2007, **56**(12)**:** 1115-1118.

3. Yang Z, Liu Q, Li J, Wang Z, Guo A, Linghu R*, et al.* Lattice instability of V_2AlC at high pressure. *Science China Physics, Mechanics and Astronomy* 2013, **56**(5)**:** 916-924.

4. Pietzka M, Schuster J. Summary of constitutional data on the aluminum-carbon-titanium system. *Journal of Phase Equilibria* 1994, **15**(4)**:** 392-400.

5. Barsoum M, El-Raghy T, Rawn C, Porter W, Wang H, Payzant E*, et al.* Thermal properties of Ti3SiC2. *Journal of Physics and Chemistry of Solids* 1999, **60**(4)**:** 429-439.

6. Rawn C, Barsoum M, El-Raghy T, Procipio A, Hoffmann C, Hubbard C. Structure of Ti_4AlN_3—a layered $M_{n+1}AX_n$ nitride. *Materials research bulletin* 2000, **35**(11)**:** 1785-1796.

7. Hu C, Li F, Zhang J, Wang J, Wang J, Zhou Y. Nb4AlC3: A new compound belonging to the MAX phases. *Scripta Materialia* 2007, **57**(10)**:** 893-896.

8. Holland T, Redfern S. Unit cell refinement from powder diffraction data: the use of regression diagnostics. *Mineralogical Magazine* 1997, **61**(1)**:** 65-77.

9. Jiang C-C, Goto T, Hirai T. Non-stoichiometry of titanium nitride plates prepared by chemical vapour deposition. *Journal of alloys and compounds* 1993, **190**(2)**:** 197-200.

10. Pflüger J, Fink J, Weber W, Bohnen KP, Crecelius G. Dielectric properties of TiC_x, TiN_x, VC_x, and VN_x from 1.5 to 40 eV determined by electron-energy-loss spectroscopy. *Physical Review B* 1984, **30**(3)**:** 1155-1163.

11. Exner H. Physical and chemical nature of cemented carbides. *International metals reviews* 1979, **24**(1)**:** 149-173.

12. Toby BH, Von Dreele RB. GSAS‐II: the genesis of a modern open‐source all purpose crystallography software package. *Journal of Applied Crystallography* 2013, **46**(2)**:** 544-549.

13. Shang S, Wang Y, Liu Z-K. First-principles elastic constants of α-and θ-Al 2 O 3. *Applied Physics Letters* 2007, **90**(10)**:** 101909.

14. Hu Y-J, Shang S-L, Wang Y, Darling KA, Butler BG, Kecskes LJ*, et al.* Effects of alloying elements

and temperature on the elastic properties of W-based alloys by first-principles calculations. *Journal of Alloys and Compounds* 2016, **671:** 267-275.

15. Simmons G, Wang H. Single crystal elastic constants and calculated aggregate properties: A handbook 2nd ed., 370. MIT Press, Cambridge, Mass; 1971.

16. Mouhat F, Coudert F-X. Necessary and sufficient elastic stability conditions in various crystal systems. *Physical Review B* 2014, **90**(22)**:** 224104.
